# Should manufacturers choose technological innovation in dual-channel supply chains during emergencies?

**DOI:** 10.1371/journal.pone.0327014

**Published:** 2025-07-03

**Authors:** Yuting Zhang, Juan Shang

**Affiliations:** School of Economics & Management, Xidian University, Xi’an, Shaanxi, China; Harbin Institute of Technology, CHINA

## Abstract

This study investigates pricing and coordination strategies for a dual-channel supply chain (DCSC), considering technological innovations in emergencies. We have established the DCSC model consisting of a manufacturer, a retailer, and an E-commerce platform (ECP). Whether manufacturers choose to invest in technological innovation during emergencies can be divided into traditional production mode and technological innovation mode. Using the reverse induction method to solve the Stackelberg game problem, explore the pricing and channel selection strategies of each member in a DCSC under different modes. In addition, a revenue-sharing contract for a DCSC under emergencies was designed and improved. Research has shown that under emergencies, consumers’ technological innovation preference can increase the profits of each member in the DCSC and manufacturers’ technological innovation level. Manufacturers are more willing to choose technological innovation mode rather than traditional production mode. However, an increase in the commission rate of ECP can hinder the level of technological innovation of manufacturers and affect the issue of choosing between offline channel and ECP channel. Specifically, when the commission rate exceeds a certain threshold, the offline channel should be chosen. Finally, traditional revenue-sharing contracts fail to effectively coordinate DCSC that incorporate technological innovation during emergencies. To address this limitation, an improved revenue-sharing contract is proposed, which enhances the level of technological innovation while achieving Pareto improvements within the DCSC.

## 1 Introduction

The occurrence of major emergencies brings great challenges to social stability and the development of the global economy and seriously threatens the personal and property safety of the general public. In 2005, Hurricane Katrina in the United States caused economic losses of up to $200 billion. The 2008 Wenchuan earthquake in China caused significant losses to the local area. In 2011, when the massive earthquake and tsunami in Japan caused a leak at the Fukushima Daiichi nuclear power plant, Toyota’s parts suppliers were unable to deliver parts in the quantities and at the speeds expected, forcing Toyota to shut down production for several days [1]. Although the probability of occurrence of these natural disasters is low, when they do occur, the impact is enormous [2–4]. The supply chain will also encounter more new problems and challenges [5].

Faced with the impact of unexpected events and technological advancements, consumers will learn to cope with various innovative ways, and new shopping habits and preferences will also emerge [6]. In August 2023, Japan initiated the discharge of nuclear-contaminated water into the sea, and the General Administration of Customs of the People’s Republic of China announced on the same day to impose a total suspension on the import of aquatic products of Japanese origin [7]. Due to the possible hazards of seafood, Chinese consumers have also developed certain preferences in their commodity purchasing choices to protect their health. In the face of this unexpected event, consumers have a stronger preference for alternative, innovative products. For example, in Xinjiang, new technological innovations have transformed resources such as saline land into “synthetic seawater” suitable for seafood farming. “Xinjiang seafood” is favored and loved by consumers, bringing more profits to aquaculture enterprises. The outbreak of the COVID-19 epidemic and its rapid spread around the world have had a serious impact on the stable operation of society [8]. At the beginning of the COVID-19 epidemic, the demand for masks increased sharply [9]. China’s large automobile brand Wuling Group has innovated its mask production equipment and independently produced masks, effectively alleviating the problem of mask shortage. However, technological innovation requires a large amount of capital investment, especially in a volatile environment, will the cost of that investment result in losses for the enterprise? Some conservative firms have continued their traditional production methods to avoid the financial pressure of technological innovation.

When there are emergencies such as supply chain disruptions, governments and related enterprises of various countries quickly take response measures to maximize the protection of people’s lives and minimize the impact of emergencies on economic and social development [10]. In May 2019, the US Department of Commerce included Huawei and its related companies in the “Entity List”. Faced with sudden supply disruptions, members of the supply chain and consumers have a clear understanding of the importance of technological innovation. This also profoundly demonstrates that technological innovation can effectively improve the resilience of supply chains in turbulent environments [11]. It also enhances consumer preference for technologically innovative products. Subsequently, Huawei has actively invested in the field of chip self-research and has achieved the goal of independent manufacturing by independently developing a variety of chips. In September 2023, Huawei Mate60 series smartphones broke through the blockade and were launched with Kirin chips, attracting consumers’ rush to buy and praise.

In addition, when emergencies occur, upstream and downstream supply chain firms often resort to alliances to cope with uncertain external changes and impacts. At the initial stage of the outbreak of the COVID-19, BAIC Group took active measures to take the lead in establishing a special fund pool of nearly 1 billion yuan to create a business model of a “community of shared future” for manufacturers to jointly respond to the possible losses caused by the COVID-19. Therefore, coordinating among supply chain members in the event of emergencies and striving for maximum profit is also one of the key considerations and research issues.

In the context of emergencies, ECP emerges as the most accessible shopping channel for consumers. Given the myriad challenges that emergencies impose on supply chain systems, developing a supply chain model that integrates technological innovation to mitigate uncontrollable factors is imperative. However, both academic literature and practical applications frequently overlook the potential impacts of emergencies on supply chains. Therefore, based on real-world conditions, we have developed a DCSC model for emergencies that comprises a manufacturer, an offline retailer, and an online ECP. Considering the inherent unpredictability of emergencies, a comprehensive analysis of parameter uncertainties is necessary to bolster the resilience and sustainability of the supply chain.

Faced with the frequent occurrence of emergencies, consumers’ shopping habits and preferences have also undergone significant changes, posing new challenges to the construction of supply chain operation and management models under emergencies. Affected by COVID-19, the traditional retail industry has been hit hard, but more and more consumers are turning to online platforms for shopping, and the online retail market is still developing rapidly [12]. According to the eMarketer report, global retail e-commerce sales will reach $5.82 trillion in 2023, a year-on-year increase of 10%, with a retail e-commerce penetration rate of 19.40%. Meanwhile, the ECP business model is favored by consumers, and more and more manufacturers are opening ECP channels, such as Apple, Procter & Gamble, Gree, and other manufacturers. The innovation of the supply chain usually relies on platform economy to fundamentally solve the operational mode of the supply chain [13]. In 2023, there will be a sudden extremely heavy rainstorm in the Beijing-Tianjin-Hebei region of China, and about 4 million books will be drowned and discarded. To reduce losses, BooksChina.com launched innovative marketing methods to save itself, selling 99 yuan “BooksChina.com encourage parcel” on E-commerce platforms (ECPs) such as Taobao and JD.com. Consumers express their love by purchasing instead of donating. In the face of emergencies, as the ECP is the most accessible shopping channel for consumers. Therefore, relying on the real situation, we construct a DCSC model based on one manufacturer, one retailer, and one ECP close to reality.

Based on the above background, this study attempts to answer the following questions:

1. When an emergency occurs, does technological innovation by a manufacturer affect the profitability of each member of the DCSC compared to a conservative manufacturer using traditional production methods?
2. Will consumers’ preference for technological innovation improve manufacturers’ level of technological innovation and the profits of each member of the DCSC?
3. How does the commission rate of ECP affect the level of technological innovation of manufacturers and the profits of each member of the DCSC?
4. Is there a coordination contract suitable for technological innovation in the DCSC, which enables supply chain members to achieve Pareto improvement in the event of emergencies?

Although the impact of emergencies on supply chains has garnered increasing scholarly attention, relatively few studies have proposed solutions to these challenges. This research aims to fill this gap. Firstly, drawing on real-world conditions and Hu et al. [14], we develop a dual-channel model comprising a manufacturer, an offline brick-and-mortar retailer, and an ECP. Our study comprehensively examines the response measures that the DCSC should adopt following emergencies. We analyze and compare the optimal pricing and coordination strategies of the dual-channel model under both traditional production and technological innovation modes in the aftermath of emergencies. Secondly, the impact of consumer technological innovation preferences and ECP commission rate on the level of technological innovation and the profits of each member of the supply chain were considered. Finally, a coordination contract for the DCSC that considers technological innovation in the event of emergencies was designed. Provide more effective operational strategies and management insights for the supply chain in the event of emergencies.

The rest of the paper is structured as follows. Section 2 reviews the relevant literature. Section 3 introduces the model assumptions and parameter settings. Section 4 establishes DCSC models for traditional production mode and technological innovation mode under emergencies, and analyzes and compares them. Section 5 designs a coordination contract suitable for DCSC under emergencies. Section 6 summarizes the relevant findings of this paper and provides managerial insights for managers.

## 2 Relevant literature

Our research mainly involves three aspects. The first part is the research on operational decision-making under emergencies, the second part is the research on supply chain innovation, and the third part is the research on DCSC.

### 2.1 Operational decision-making during emergencies

The occurrence of emergencies has a significant impact on the stable operation of society and the productive life of the population. After the outbreak of the COVID-19 epidemic, scholars regard the study of public health emergencies as a research focus. Public opinion research on emergencies has been widely studied, including information disclosure [15], dissemination of erroneous information [16], and government media participation [17]. The public’s response to emergencies is also an area worthy of in-depth research. Li et al. [18] examined the impact of public emotions such as anger, anxiety, and sadness on the scale of social media posts following a natural disaster. Song et al. [19] explored the impact of external stimuli related to the COVID-19 epidemic on public mood. Emergencies are bound to have a direct or indirect impact on the population, and mood changes affect many aspects. In addition to the negative impact of public emotions, is there a positive impact, how to explore positive effects, and how to maximize positive effects? All of the above issues require more in-depth study.

The emotional fluctuations of the public in the face of emergencies not only affect the dissemination of event information but also have a crucial impact on the operation of the supply chain through their purchasing and consumption preferences [20]. Consumer purchasing preferences are changed by environmental changes caused by unexpected events, and some consumers have different purchasing preferences due to the management methods of enterprises. Eger et al. [21] studied the changes in consumer behavior patterns during the COVID-19 pandemic in the Czech Republic using a questionnaire survey method. Pani et al. [22] evaluated the issue of consumer shopping preferences during the COVID-19 pandemic using a sample of 483 consumers in Portland, studied the public’s acceptance of automated delivery robots, and proposed relevant guidance recommendations. Most of the above studies on emergencies are based on empirical research, analyzing the data collected during or after the event, and focusing on more macroscopic research areas such as emergency management decisions and information dissemination in emergencies.

Since the involvement of multiple enterprises and consumers in the production and service of the supply chain, the occurrence of emergencies will inevitably affect the smooth operation of the supply chain and the shopping experience of consumers. Therefore, the production operation of the supply chain during emergencies is a hot issue that scholars continue to study. Balcik and Ak [23] studied the supplier selection problem in a supply chain model consisting of one rescue organization with multiple suppliers after the establishment of a contractual contract. Torabi et al. [24] focused on the procurement of emergency supplies. Guru et al. [25] used a mixed method of qualitative and quantitative analysis to study the issues related to the distribution channel selection of the supply chain of India’s FMCG industry after the COVID-19 epidemic turned consumer preferences to digital platforms. Shen and Sun [26] used JD as a case to study the response measures of the supply chain of ECPs to COVID-19 and proposed advanced technologies such as automation and the importance of collaboration. Baz and Ruel [27] used empirical research methods to propose that when the supply chain encounters unexpected situations such as interruptions, relationship governance should be carried out with supply chain members. We also included consumer preferences in the scope of our research and studied the coordination issues among supply chain members from the perspective of supply chain models.

### 2.2 Supply chain innovation

Supply chain innovation has received a great deal of attention from scholars, but research in the field of supply chain innovation is fragmented [28]. Most scholars have focused on examining the impact of innovation on supply chain resilience [11,^29^,^30^], risk management capabilities [31,32], financial constraints [33], financial subsidies [34,35], and cooperative relationships among supply chain members [36]. Silvestre [37] suggests the importance of supply chain innovation in environmentally turbulent environments through an in-depth case study of supply chains in the upstream oil and gas industry in Brazil. Chatterjee et al. [38] explored the impact of big data-driven innovation in small and medium-sized enterprises on their supply chain systems in the post-COVID-19 scenario. Liu et al. [39] explored the influencing factors of corporate smart supply chain innovation based on several case studies. Howell [40] studied the relationship between corporate innovation and survival using Chinese entrepreneurial enterprises as samples, and the results showed that innovation can increase the survival probability of enterprises. Al-Omoush et al. [41] collected empirical data from key industries and explored the relationship between e-supply chain collaboration, collaborative innovation, supply chain agility, and value co-creation. Most of the above studies are based on specific cases and mainly study supply chain innovation from a macro perspective. In contrast, our study focuses on supply chain innovation using a game theory approach.

Although, there are a few scholars who have studied supply chain innovation in detail using the game theory approach. Krishnan et al. [42] used a sequential game theory approach to analyze the investment decision problem with objectives ranging from investment in innovation to market coverage to profit. Wu et al. [35] constructed an R&D cooperation game model and investigated the contractual design of the government to encourage innovation through selective subsidies. Differently, this article focuses more on the research of technological innovation in the supply chain. Technological innovation plays a vital role in supply chain operations [43,44]. Some scholars have also researched technological innovation. Radicic and Petković [45] explored the impact of digitalization on technological innovation in German SMEs. Mensah et al. [46] examined the impact of technological innovation on green growth in 28 economies of the OECD over the period 2000–2014. Although the above research involves innovation in supply chain technology, it mainly focuses on empirical research on supply chain technological innovation. However, some scholars have also established supply chain models and considered the impact of technological innovation on the supply chain. Reimann et al. [47] investigated the relationship between remanufacturing and cost reduction through technological innovation in supply chains composed of manufacturers and retailers. However, to the best of our knowledge, there is little literature that examines the impact of considering consumer preferences on supply chain technological innovation under emergencies. To fill the existing research gap, this paper studies the impact of emergencies from the perspective of supply chain management, and it is a wise choice for manufacturers to actively seek technological innovation.

### 2.3 DCSC

Another type of research related to this paper is mainly related to the channel structure of the supply chain. With the rapid development of society and the rise of mobile payment, online shopping mode is widely accepted and applied by consumers. The research on supply chains has developed from the early single traditional channel supply chain to a research model with dual channels as the mainstream [48,49]. At present, research on supply chain response to emergencies mainly focuses on risk avoidance in advance. Liu et al. [50] investigated the optimal pricing strategy for a DCSC concerning risk aversion under both complete trust and information asymmetry. Xu et al. [51] proposed an analytical framework for marking price decisions in risk-averse centralized and decentralized DCSC and analyzed the impact of risk tolerance on the pricing decisions of manufacturers and retailers. Zhu et al. [52] proposed a decision model using the CVaR criterion as a risk assessment metric and verified that a joint contract of revenue-sharing contract and repurchase contract can coordinate a DCSC.

However, current research on dual channels under the influence of supply chain risk focuses on a direct marketing dual-channel model consisting of one manufacturer and one retailer. The DCSC model, which consists of one manufacturer, one retailer, and one ECP, is closer to reality. The study of incorporating ECPs into dual channels has also been gradually focused on by scholars, but most scholars have mainly focused on channel encroachment as well as the study of comparing multiple dual-channel models [53,54]. Hu et al. [14] studied the interaction between the strategy of suppliers introducing a marketplace channel and the sales model selection of e-tailer. Zhang et al. [55] investigated the optimal operating strategies of a platform supply chain with a manufacturer and an ECP in the wholesale and platform modes. However, the focus of our research is on the post-event response strategies for emergencies based on the DCSC model.

For clarity, Table 1 summarizes the most relevant literature. Based on the above research in related literature and the problems shown in real cases, this paper makes up for the lack of existing dual-channel studies under emergencies that include ECPs. Hu et al. [14] and Zhang et al. [55] conducted related studies on supply chain structures by examining sales mode strategies in dual-channel systems. However, our work differs in several key respects. First, we focus on the response measures and channel selection strategies adopted by supply chain members in the face of emergencies. Second, we investigate the impact of technological innovation on channel selection under these circumstances. We incorporated ECPs into a DCSC model and analyzed and compared the impact of various influencing factors such as consumer technological innovation preferences, manufacturer technological innovation costs, and ECP commission rate on traditional production mode and technological innovation mode. We conducted a detailed study on pricing and technological innovation strategies among members in the dual channel under emergencies.

**Table 1:** Comparisons between this study and related literature.

Authors	Supply chain innovation	Emergencies	Dual-channel	ECP	Coordination	Consumer preference
Hu et al. [14]			√	√		
Balcik and Ak [23]		√			√	
Guru et al. [25]		√		√		
Chatterjee et al. [38]	√	√				
Al-Omoush et al. [41]	√			√	√	
Wu et al. [35]	√				√	
Liu et al. [50]		√	√			
Xu et al. [51]			√		√	
Zhu et al. [52]		√	√		√	
Zhang et al. [55]			√	√		√
This paper	√	√	√	√	√	√

Furthermore, our analysis demonstrates that traditional revenue-sharing contracts are insufficient for effectively coordinating DCSC that incorporate technological innovation. To address this shortcoming, we develop and refine a revenue-sharing contract that not only enhances technological innovation but also achieves Pareto improvements for DCSC, thereby providing both a theoretical framework and practical management insights for supply chain operations in the context of emergencies.

## 3 Model description and assumptions

**Fig 1:**
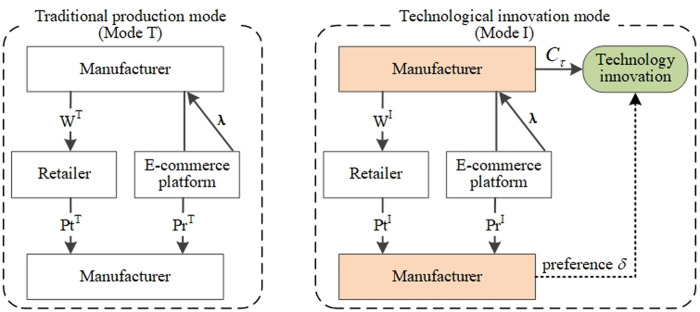
Two modes are considered in the dual channels supply chain.

This paper considers whether manufacturers should engage in technological innovation in a DCSC that introduces ECP channels during emergencies. And pricing and coordination strategies for traditional production mode and technological innovation mode. In the traditional production mode, manufacturers are more conservative and choose to adopt traditional production methods to avoid the high cost of technological innovation that cannot be recovered in case of emergencies. In addition, manufacturers open both offline channels and online ECP channels and cooperate with ECPs by paying reseller commissions to sell on ECPs. It is worth noting that the commission rate is set by the ECP, while the retail price of the ECP is decided by the manufacturer [55]. Furthermore, some manufacturers proactively invest in technological innovation during emergencies. A notable example is GE Healthcare [56], which, during the COVID-19 pandemic, allocated substantial resources to accelerate the development and improvement of ventilator equipment in order to address the urgent needs of the healthcare system. Under the technological innovation mode, manufacturers actively counteract the effects of emergencies relative to the traditional production mode and cater to consumers’ preference for technological innovation by investing in related costs [28,38]. The DCSC models for both scenarios are presented in Fig 1.

The Parameter definitions are shown in Table 2. In the creation of the model, we made several assumptions.

**Table 2:** Parameter definition.

Parameters	Definition
a	Total market potential
γ	The coefficient of cross-price sensitivity
λ	The commission rate
$\tau^{J}$	The level of technological innovation in Mode I, where J∈{I,IC,Co}, IC (Centralized decision-making), Co (Contract)
δ	The coefficient of consumers’ technological innovation preference
k	The coefficient of technological innovation cost
$C_{\tau }$	The technological innovation cost
$W^{J}$	Wholesale price in Mode J, where J∈{T,I}
$P_{i}^{J}$	Retail price of i in Mode J, where i∈{r,e}, r (retailer), e (ECP), J∈{T,I}
$D_{i}^{J}$	Market demand of i in Mode J, where i∈{r,e}, J∈{T,I}
$\prod_{i}^{J}$	The profit of i in Mode J, where i∈{r,e,m}, m (manufacturer), J∈{T,I}
$\prod^{J}$	The profit of the whole supply chain in Mode J, J∈{T,I}

Due to the unpredictable nature of emergencies, which often affect various aspects of existing supply chain operations, consumer preferences may shift, showing a greater inclination toward products featuring technological innovation. To mitigate potential losses and cater to these evolving preferences, some manufacturers invest in technology. However, given the high costs associated with technological innovation and the risk of failure, others continue to adopt the traditional production mode after emergencies to avoid losses from unsuccessful innovations. The primary objective of this study’s mode is to compare the strategies of DCSC in adopting technological innovation during emergencies and to further investigate its impact on each supply chain member. Based on real-world conditions and with a focus on the research problem at hand, we propose the following assumptions:

**Assumption 1.** For this analysis, it is assumed that the cross-channel price sensitivity is symmetric and that sales volumes are weakly neutral concerning the cross-price sensitivity, $0<\gamma <\frac{1}{4}$ [50–52].

**Assumption 2.** In practice, ECPs such as JD.com [57], Taobao [58], and Amazon [59] charge fixed technical service fee rates and transaction service fee rates based on different product categories. Therefore, this paper uniformly refers to the commission rate and assumes that λ is an exogenous variable, $0<\lambda <\frac{1}{2}$ [60].

**Assumption 3.** For production costs, to ensure that the analytical solution for each member of the DCSC model is positive, this paper assumes $k>k_{0}$, $k_{0}=\frac{\delta^{2}}{1-\gamma }$ [61].

**Assumption 4.** We assume that the cost of technological innovation paid by manufacturers in the event of an emergency is $C_{\tau }$, $C_{\tau }=\frac{1}{2}k\tau^{2}$ [62]. Due to the complexity of the model, only the cost of technological innovation paid by the manufacturer is considered and the manufacturer’s cost of production is assumed to be zero [63]. In addition, the situation where retail prices increase significantly due to emergencies is not considered, and there is no fraudulent behavior among members of the supply chain.

## 4 Model development

### 4.1 Traditional production mode (Mode T)

**Fig 2:**
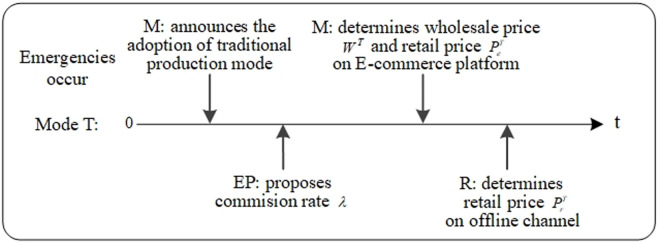
Event sequence of Mode T.

In traditional production mode, even in the event of emergencies, manufacturers still choose traditional production mode. The event sequence of the manufacturer, the retailer, and the ECP is in Fig 2. The manufacturer determines the wholesale price $W^{T}$ and retail price $P_{e}^{T}$ on the ECP. Then, the retailer determines the retail price $P_{r}^{T}$ on the offline channel.

In mode T, we have the demand functions for retailer and ECP as follows:


$$D_{r}^{T}=a-P_{r}^{T}+\gamma P_{e}^{T}$$ (1)

$$D_{t}^{T}=a-P_{t}^{T}+\gamma P_{e}^{T}$$ (2)

The profit functions for the retailer, the ECP, the manufacturer, and the whole supply chain are as follows:


$$\begin{array}{c} \underset{P_{r}^{T}}{\max\prod {\mathrm{\backslash nolimits}}_{r}^{T}}=(P_{r}^{T}-W^{T})D_{r}^{T} \end{array}$$ (3)

$$\begin{array}{c} \underset{\left(W^{T},P_{e}^{T}\right)}{\max\prod {\mathrm{\backslash nolimits}}_{m}^{T}}=W^{T}D_{r}^{T}+(1-\lambda )P_{e}^{T}D_{e}^{T} \end{array}$$ (4)

$$\prod {\mathrm{\backslash nolimits}}_{e}^{T}=\lambda P_{e}^{T}D_{e}^{T}$$ (5)

$$\prod {\mathrm{\backslash nolimits}}^{T}=P_{r}^{T}D_{r}^{T}+P_{e}^{T}D_{e}^{T}$$ (6)

Equilibrium outcomes of Mode T are obtained by solving the problem by backward induction.

**Lemma 1.** In Mode T, there exist equilibrium solutions and profits:

(i) Retail prices and wholesale price

$$P_{r}^{T}=\frac{a\left(1-\lambda \right)\left(6+4\gamma -2\gamma^{2}-\gamma \lambda \right)}{8\left(1-\gamma^{2}\right)\left(1-\lambda \right)-\gamma^{2}\lambda^{2}},$$

$$P_{e}^{T}=\frac{a\left(4+4\gamma -4\lambda -3\gamma \lambda \right)}{8\left(1-\gamma^{2}\right)\left(1-\lambda \right)-\gamma^{2}\lambda^{2}},$$

$$W^{T}=\frac{a\left(1-\lambda \right)\left(4+4\gamma -2\gamma \lambda -\gamma^{2}\lambda \right)}{8\left(1-\gamma^{2}\right)\left(1-\lambda \right)-\gamma^{2}\lambda^{2}};$$

(ii) Market demand

$$D_{r}^{T}=\frac{a\left(1+\gamma \right)\left(1-\lambda \right)\left(2-2\gamma +\gamma \lambda \right)}{8\left(1-\gamma^{2}\right)\left(1-\lambda \right)-\gamma^{2}\lambda^{2}},$$

$$D_{e}^{T}=\frac{a\left(1+\gamma \right)\left(2\left(2-\gamma^{2}\right)\left(1-\lambda \right)+\gamma \lambda -2\gamma \right)}{8\left(1-\gamma^{2}\right)\left(1-\lambda \right)-\gamma^{2}\lambda^{2}};$$

(iii) The profit of supply chain members

$$\prod {\mathrm{\backslash nolimits}}_{r}^{T}=\frac{a^{2}{\left(1+\gamma \right)}^{2}{\left(1-\lambda \right)}^{2}{\left(2-2\gamma +\gamma \lambda \right)}^{2}}{{\left(8\left(1-\gamma^{2}\right)\left(1-\lambda \right)-\gamma^{2}\lambda^{2}\right)}^{2}},$$

$$\prod {\mathrm{\backslash nolimits}}_{e}^{T}=\frac{a^{2}\left(1+\gamma \right)\lambda \left(4+4\gamma -4\lambda -3\gamma \lambda \right)\left(2\left(2-\gamma^{2}\right)\left(1-\lambda \right)+\gamma \lambda -2\gamma \right)}{{\left(8-8\lambda -\gamma^{2}\left(8-8\lambda +\lambda^{2}\right)\right)}^{2}},$$

$$\prod {\mathrm{\backslash nolimits}}_{m}^{T}=\frac{a^{2}\left(1+\gamma \right)\left(1-\lambda \right)\left(3+\gamma -2\lambda -\gamma \lambda \right)}{8\left(1-\gamma^{2}\right)\left(1-\lambda \right)-\gamma^{2}\lambda^{2}},$$

(iv) The profit of the whole supply chain

$$\prod {\mathrm{\backslash nolimits}}^{T}=\frac{a^{2}\left(1+\gamma \right)\left(4{\left(1-\lambda \right)}^{2}\left(7+\gamma +\gamma \lambda \right)-2\gamma^{3}\left(1-\lambda \right)\left(2-\lambda^{2}\right)-\gamma^{2}\left(28-56\lambda +36\lambda^{2}-8\lambda^{3}+\lambda^{4}\right)\right)}{{\left(8-8\gamma^{2}-8\lambda +8\gamma^{2}\lambda -\gamma^{2}\lambda^{2}\right)}^{2}}.$$

### 4.2 Technological innovation mode (Mode I)

**Fig 3:**
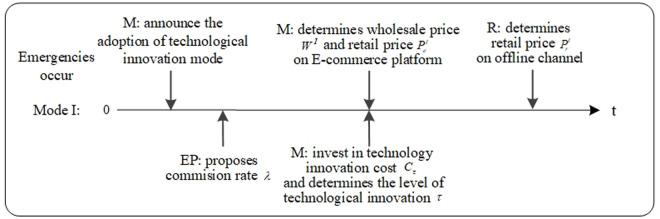
Event sequence of Mode I.

In technological innovation mode, the manufacturer takes the initiative to invest in technological innovation due to changes in the external environment caused by emergencies. The event sequence of the manufacturer, the retailer, and the ECP is in Fig 3. The manufacturer determines wholesale price $W^{I}$, retail price $P_{e}^{I}$ on the ECP, and the level of technological innovation $\tau^{I}$. Then, the retailer determines the retail price $P_{r}^{I}$ on the offline channel.

In mode I, we have the demand functions for retailer and ECP as follows:


$$\begin{array}{c} D_{r}^{I}=a-P_{r}^{I}+\gamma P_{e}^{I}+\delta \tau^{I} \end{array}$$ (7)

$$D_{e}^{I}=a-P_{e}^{I}+\gamma P_{r}^{I}+\delta \tau^{I}$$ (8)

The profit functions for the retailer, the ECP, the manufacturer, and the whole supply chain are as follows:


$$\begin{array}{c} \underset{P_{r}^{I}}{\max\prod {\mathrm{\backslash nolimits}}_{r}^{I}}=\left(P_{r}^{I}-W^{I}\right)D_{r}^{I} \end{array}$$ (9)

$$\underset{\left(W^{I},P_{e}^{I},\tau^{I}\right)}{\max\prod {\mathrm{\backslash nolimits}}_{m}^{I}}=W^{I}D_{r}^{I}+\left(1-\lambda \right)P_{e}^{I}D_{e}^{I}-\frac{1}{2}k{\left(\tau^{I}\right)}^{2}$$ (10)

$$\prod {\mathrm{\backslash nolimits}}_{e}^{I}=\lambda P_{e}^{I}D_{e}^{I}$$ (11)

$$\prod {\mathrm{\backslash nolimits}}^{I}=P_{r}^{I}D_{r}^{I}+P_{e}^{I}D_{e}^{I}-\frac{1}{2}k{\left(\tau^{I}\right)}^{2}$$ (12)

Equilibrium outcomes of Mode I are obtained by solving the problem by backward induction.

**Lemma 2.** In Mode I, there exist equilibrium solutions and profits:

(i) Retail prices and wholesale price

$$P_{r}^{I}=\frac{ak\left(1-\lambda \right)\left(6+4\gamma -2\gamma^{2}-\gamma \lambda \right)}{2\left(1+\gamma \right)\delta^{2}\left(1-\lambda \right)\left(2\lambda +\gamma \lambda -3-\gamma \right)+k\left(8-8\gamma^{2}-8\lambda +8\gamma^{2}\lambda -\gamma^{2}\lambda^{2}\right)},$$

$$P_{e}^{I}=\frac{ak\left(4+4\gamma -4\lambda -3\gamma \lambda \right)}{-2\left(1+\gamma \right)\delta^{2}\left(1-\lambda \right)\left(3+\gamma -2\lambda -\gamma \lambda \right)+k\left(8-8\gamma^{2}-\left(8-8\gamma^{2}\right)\lambda -\gamma^{2}\lambda^{2}\right)},$$

$$W^{I}=\frac{ak\left(1-\lambda \right)\left(4+4\gamma -2\gamma \lambda -\gamma^{2}\lambda \right)}{2\left(1+\gamma \right)\delta^{2}\left(1-\lambda \right)\left(-3-\gamma +\left(2+\gamma \right)\lambda \right)+k\left(8-8\gamma^{2}-\left(8-8\gamma^{2}\right)\lambda -\gamma^{2}\lambda^{2}\right)};$$

(ii) The level of technological innovation in Mode I

$$\tau^{I}=\frac{2a\left(1+\gamma \right)\delta \left(1-\lambda \right)\left(3+\gamma -2\lambda -\gamma \lambda \right)}{2\left(1+\gamma \right)\delta^{2}\left(1-\lambda \right)\left(-3-\gamma +\left(2+\gamma \right)\lambda \right)+k\left(8-8\gamma^{2}-\left(8-8\gamma^{2}\right)\lambda -\gamma^{2}\lambda^{2}\right)};$$

(iii) Market demand

$$D_{r}^{I}=\frac{ak\left(1+\gamma \right)\left(1-\lambda \right)\left(2-2\gamma +\gamma \lambda \right)}{2\left(1+\gamma \right)\delta^{2}\left(1-\lambda \right)\left(-3-\gamma +\left(2+\gamma \right)\lambda \right)+k\left(8-8\gamma^{2}-\left(8-8\gamma^{2}\right)\lambda -\gamma^{2}\lambda^{2}\right)},$$

$$D_{e}^{I}=\frac{ak\left(1+\gamma \right)\left(-2\gamma +2\left(-2+\gamma^{2}\right)\left(-1+\lambda \right)+\gamma \lambda \right)}{2\left(1+\gamma \right)\delta^{2}\left(1-\lambda \right)\left(-3-\gamma +\left(2+\gamma \right)\lambda \right)+k\left(8-8\gamma^{2}-8\lambda +8\gamma^{2}\lambda -\gamma^{2}\lambda^{2}\right)};$$

(iv) The profit of supply chain members

$$\prod {\mathrm{\backslash nolimits}}_{r}^{I}=\frac{a^{2}k^{2}{\left(1+\gamma \right)}^{2}{\left(1-\lambda \right)}^{2}{\left(2-2\gamma +\gamma \lambda \right)}^{2}}{{\left(2\left(1+\gamma \right)\delta^{2}\left(1-\lambda \right)\left(-3-\gamma +\left(2+\gamma \right)\lambda \right)+k\left(8-8\gamma^{2}-\left(8-8\gamma^{2}\right)\lambda -\gamma^{2}\lambda^{2}\right)\right)}^{2}}$$

$$\prod {\mathrm{\backslash nolimits}}_{e}^{I}=\frac{a^{2}k^{2}\left(1+\gamma \right)\lambda \left(4+4\gamma -4\lambda -3\gamma \lambda \right)\left(4-2\gamma -2\gamma^{2}-4\lambda +\gamma \lambda +2\gamma^{2}\lambda \right)}{{\left(2\left(1+\gamma \right)\delta^{2}\left(1-\lambda \right)\left(-3-\gamma +\left(2+\gamma \right)\lambda \right)+k\left(8-8\gamma^{2}-\left(8-8\gamma^{2}\right)\lambda -\gamma^{2}\lambda^{2}\right)\right)}^{2}},$$

$$\prod {\mathrm{\backslash nolimits}}_{m}^{I}=\frac{a^{2}k\left(1+\gamma \right)\left(1-\lambda \right)\left(3+\gamma -2\lambda -\gamma \lambda \right)}{2\left(1+\gamma \right)\delta^{2}\left(1-\lambda \right)\left(-3-\gamma +\left(2+\gamma \right)\lambda \right)+k\left(8-8\gamma^{2}-\left(8-8\gamma^{2}\right)\lambda -\gamma^{2}\lambda^{2}\right)},$$

(v) The profit of the whole supply chain

$$\prod {\mathrm{\backslash nolimits}}^{I}=\frac{a^{2}k\left(1+\gamma \right)\left(\begin{array}{c} k\left(4{\left(1-\lambda \right)}^{2}\left(7+\gamma +\gamma \lambda \right)-2\gamma^{3}\left(1-\lambda \right)\left(2-\lambda^{2}\right)-\gamma^{2}\left(28-56\lambda +36\lambda^{2}-8\lambda^{3}+\lambda^{4}\right)\right)\\ -2\left(1+\gamma \right)\delta^{2}{\left(1-\lambda \right)}^{2}{\left(3+\gamma -2\lambda -\gamma \lambda \right)}^{2} \end{array}\right)}{{\left(2\left(1+\gamma \right)\delta^{2}\left(-1+\lambda \right)\left(-3+\gamma \left(-1+\lambda \right)+2\lambda \right)+k\left(8\left(-1+\lambda \right)+\gamma^{2}\left(8+\left(-8+\lambda \right)\lambda \right)\right)\right)}^{2}}$$

**Proposition 1.** (i) $\frac{\partial W^{I}}{\partial \delta }>0$, $\frac{\partial P_{r}^{I}}{\partial \delta }>0$, $\frac{\partial P_{e}^{I}}{\partial \delta }>0$;
(ii) $\frac{\partial \tau^{I}}{\partial \delta }>0$;
(iii) $\frac{\partial D_{r}^{I}}{\partial \delta }>0$, $\frac{\partial D_{e}^{I}}{\partial \delta }>0$;
(iv) $\frac{\partial \prod_{r}^{I}}{\partial \delta }>0$, $\frac{\partial \prod_{e}^{I}}{\partial \delta }>0$, $\frac{\partial \prod_{m}^{I}}{\partial \delta }>0$, $\frac{\partial \prod^{I}}{\partial \delta }>0$.

See Appendix for proof.

Proposition 1 states that the sensitivity of the DCSC in the technological innovation model concerning consumer preferences for technological innovation is analyzed as follows: (1) In terms of price, wholesale price, retail price of retailer, and retail price of ECP all increase with the increasing of consumers’ technological innovation preference. This is because in the face of emergencies and changes in the external environment of the supply chain, manufacturers respond to environmental changes promptly and make certain technological innovation efforts. At this point, consumers’ shopping mindsets and preferences will change and they will be willing to pay higher prices for manufacturer’s technological innovation. Consumers’ preference for technological innovation drives manufacturers to improve their level of technological innovation, resulting in higher costs for additional innovation efforts. The increase in costs will inevitably lead to a rise in prices. Meanwhile, consumers with a greater preference for technological innovation can also accept higher retail prices.

**Fig 4:**
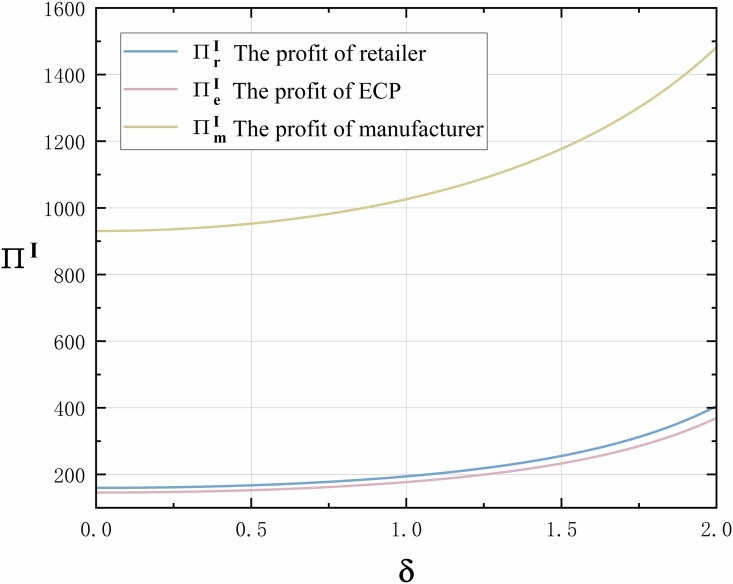
The trend of consumers’ technological innovation preference δ on supply chain member profits $\prod^{I}$ in Model I.

(2) About the level of the manufacturer’s technological innovation, when consumers’ technological innovation preference is greater, the level of the manufacturer’s technological innovation is higher. It suggests that changes in the external environment stimulate consumers’ shopping preferences when emergencies occur. The greater consumers’ preference for technological innovation and the higher their demand for innovative products, the more motivated manufacturers are to enhance their level of technological innovation.
(3) In terms of demand, the increase in consumers’ technological innovation preference can simultaneously increase the demand for offline channel and ECP channel. The impact of consumers’ technological innovation preference on demand will not change due to different channels in the supply chain.
(4) In terms of profit, according to Proposition 1 (1) and (3), consumers’ preference for technological innovation can increase retail prices and demand in both online and offline channels. Increases in retail prices and demand will inevitably increase the profits of supply chain members. Therefore, the profits of retailer, ECP, manufacturer, and the overall whole supply chain all increase with the increasing of consumers’ technological innovation preference, as shown in Fig 4. It should be noted that, in order to clearly present the results of the sensitivity analysis and the comparative conclusions, we provide several numerical examples to validate the results derived in the previous sections and to offer an intuitive reflection of the subsequent findings. To ensure that the parameters are appropriately set and all assumptions from the previous section are met, we refer to real-world cases, such as the numerical settings of major ECPs like JD.com [57], Taobao [58], and Amazon [59], as well as the parameter estimates found in related literature [64–67]. Therefore, we assume the following parameters: a=50, γ=0.2, λ=0.2, k=8. Moreover, to maintain consistency throughout the paper, the aforementioned parameters apply to all subsequent figures without further elaboration.

**Proposition 2.**(i) $\frac{\partial \tau^{I}}{\partial \lambda }<0$;
(ii) if $0<\lambda <\lambda_{P1}^{I}$, $P_{r}^{I}>P_{e}^{I}$, $D_{e}^{I}>D_{r}^{I}$;
(iii) if $\lambda_{P1}^{I}<\lambda <1$, $P_{e}^{I}>P_{r}^{I}$, $D_{r}^{I}>D_{e}^{I}$.

See Appendix for proof.

Proposition 2 indicates that in the technological innovation mode, the commission collected by the ECP comes from manufacturer. When the commission rate increases, it increases the cost of opening an ECP channel, squeezing out the cost for manufacturer to use for technological innovation. As a result, the level of technological innovation of the manufacturer decreases as the commission rate increases.

In comparing the prices and demand between offline channels and ECPs, when the commission rate exceeds a certain threshold, the retail price on ECPs will be higher than that of offline channels, while demand on ECPs will be lower than that of offline channels. In such cases, a preference for offline channels is warranted, which aligns with real-world observations. When faced with the same product and absent any channel bias, consumers invariably opt for the lower-priced channel, as lower prices lead to greater product demand.

### 4.3 Comparison analysis

**Proposition 3.** (i) $W^{I}>W^{T}$,
(ii) $P_{r}^{I}>P_{r}^{T}$,
(iii) $P_{e}^{I}>P_{e}^{T}$.

See Appendix for proof.

Proposition 3 compares wholesale price, retail price in offline channel, and retail price on ECP in traditional production mode and technological innovation mode. Since in the technological innovation mode, the manufacturer invests in technological innovation, which increases the overall cost. Therefore, both wholesale prices and retail prices in both channels have increased compared to traditional production mode.

**Proposition 4.**(i) $D_{r}^{I}>D_{r}^{T}$;
(ii) $D_{e}^{I}>D_{e}^{T}$.

See Appendix for proof.

Proposition 4 compares the demand for offline channel and ECP channel between traditional production mode and technological innovation mode. According to Proposition 3, it can be seen that the retail price of the offline channel and the retail price of the e-commerce channel in the technological innovation mode are higher than in the traditional production mode. However, considering that consumers have a preference for technological innovation in the context of emergencies, the demand for offline channel and ECP channel under technological innovation mode is greater than that under traditional production mode. This is a good illustration of the fact that even though the prices of technologically innovative products are increased under emergencies, the demand is still greater than that of traditional products. This is also well demonstrated in Proposition 1(3).

**Proposition 5.** (i) $\prod {\mathrm{\backslash nolimits}}_{r}^{I}>\prod {\mathrm{\backslash nolimits}}_{r}^{T}$,
(ii) $\prod {\mathrm{\backslash nolimits}}_{e}^{I}>\prod {\mathrm{\backslash nolimits}}_{e}^{T}$,
(iii) $\prod {\mathrm{\backslash nolimits}}_{m}^{I}>\prod {\mathrm{\backslash nolimits}}_{m}^{T}$.

See Appendix for proof.

**Fig 5:**
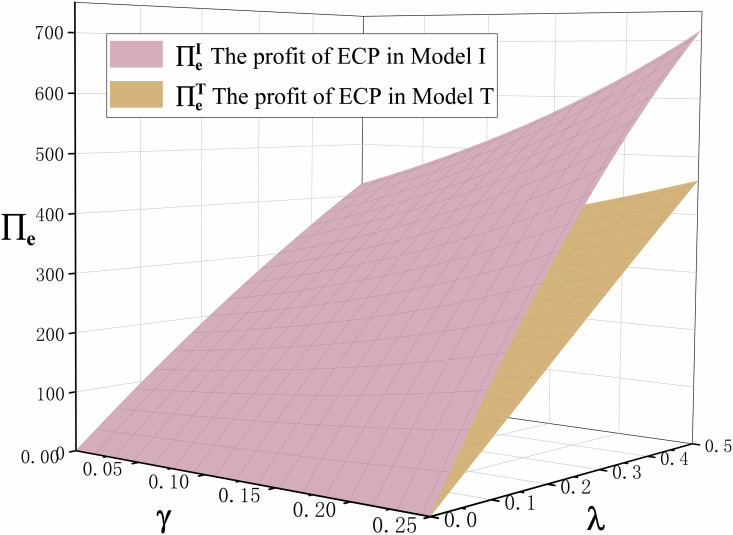
The trend of cross-price sensitivity coefficient γ and commission rate λ on ECP profits $\prod {\mathrm{\backslash nolimits}}_{e}$in Model I and Model T.

**Fig 6:**
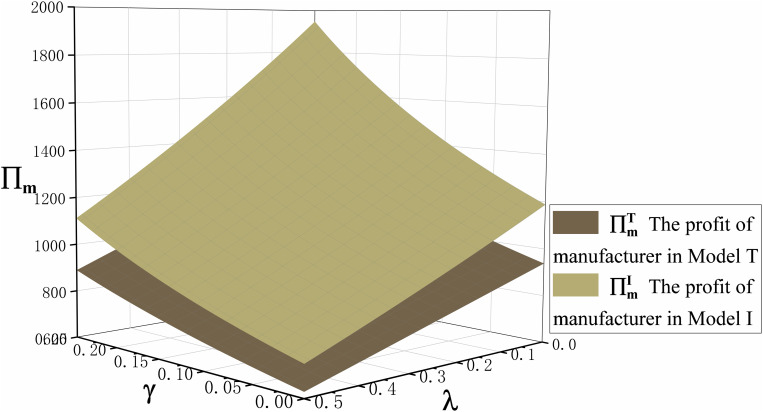
The trend of cross-price sensitivity coefficient $\begin{array}{c} \mathrm{\backslash bold}\gamma \end{array}$ and commission rate $\begin{array}{c} \mathrm{\backslash bold}\lambda \end{array}$ on manufacturers profits $\begin{array}{c} \mathrm{\backslash bold}\prod {\mathrm{\backslash nolimits}}_{m} \end{array}$ in Model I and Model T.

Proposition 5 compares the profits of retailers, ECPs, and manufacturers in the traditional production mode and the technological innovation mode, as shown in Fig 5 and Fig 6. We can clearly see the profits of retailer, ECP, and manufacturer in the technological innovation mode are greater than in the traditional production mode. This is mainly because the retail price and demand for both offline channel and ECP channel in the technological innovation mode are greater than in the traditional production mode after the emergencies. This is fully proved in Proposition 3 and Proposition 4. Therefore, when emergencies occur, manufacturers should be decisive in technological innovation.

## 5 Coordination contract

### 5.1 Centralized decision-making

When emergencies occur, upstream and downstream supply chain companies often adopt a cooperative approach to respond to changes in the environment, avoiding losses and seeking more opportunities to grow profits. This paper focuses on the coordination contract of a DCSC considering the manufacturer’s technological innovation in the event of emergencies, so the technological innovation model is selected for in-depth research. Because manufacturers and retailers always aim at maximizing their own interests under decentralized decision-making, the “double marginal effect” makes the overall profit of the supply chain not optimal under decentralized decision-making [68]. Therefore, before optimizing and coordinating the design, research should be conducted on the situation of the DCSC model in centralized decision-making under emergencies.

In centralized decision-making, the demand functions for retailer and ECP are as follows:


$$D_{r}^{IC}=a-P_{r}^{IC}+\gamma P_{e}^{IC}+\delta \tau^{IC}$$ (13)

$$D_{e}^{IC}=a-P_{e}^{IC}+\gamma P_{r}^{IC}+\delta \tau^{IC}$$ (14)

In centralized decision-making, with the premise of maximizing the overall profit of the supply chain, manufacturers and retailers jointly decide the offline channel retail price $P_{r}^{IC}$, ECP channel retail price $P_{e}^{IC}$, and technological innovation level $\tau^{IC}$. The profit function of the whole supply chain is:


$$\underset{\left(P_{r}^{IC},P_{e}^{IC},\tau^{IC}\right)}{\max\prod {\mathrm{\backslash nolimits}}^{IC}}=P_{r}^{IC}D_{r}^{IC}+P_{e}^{IC}D_{e}^{IC}-\frac{1}{2}k(\tau^{IC}{)}^{2}$$ (15)

Equilibrium outcomes of centralized decision-making are obtained by solving the problem by backward induction.

**Lemma 3.** In centralized decision-making, there exist equilibrium solutions and profits:

(i) Retail prices

$$P_{r}^{IC}=P_{e}^{IC}=\frac{ak}{2\left(1-\gamma \right)k-2\delta^{2}};$$

(ii) The level of technological innovation

$$\tau^{IC}=\frac{a\delta }{\left(1-\gamma \right)k-\delta^{2}}$$

(iii) Market demand

$$D_{r}^{IC}=D_{e}^{IC}=\frac{a\left(1-\gamma \right)k}{2\left(1-\gamma \right)k-2\delta^{2}};$$

(iv) The profit of the whole supply chain

$$\prod {\mathrm{\backslash nolimits}}^{IC}=\frac{a^{2}k}{2\left(1-\gamma \right)k-2\delta^{2}}.$$

**Proposition 6.** (i) if $k_{0}<k<k_{t}$, $P_{r}^{IC}>P_{r}^{I}$, $P_{e}^{IC}>P_{e}^{I}$;
(ii) if $k_{t}<k<k_{r}$, $P_{r}^{IC}<P_{r}^{I}$, $P_{e}^{IC}>P_{e}^{I}$;
(iii) if $k_{r}<k$, $P_{r}^{IC}<P_{r}^{I}$, $P_{e}^{IC}<P_{e}^{I}$.

See Appendix for proof.

Proposition 6 indicates that the coefficient of technological innovation cost under emergencies directly affects the retail price of offline channel and ECP channel under different decision-making. This finding is consistent with the results presented by Ghosh and Shah [69] and Zhou and Ye [70]. When the coefficient of technological innovation cost is in different intervals, there are different relationships between offline channel retail prices and ECP channel retail prices for centralized and decentralized decision-making. This is because the decision objectives differ in a DCSC: under centralized decision-making, all decisions aim to maximize the overall supply chain profit, whereas under decentralized decision-making, each supply chain member seeks to maximize its own profit [71]. Moreover, the technological innovation cost coefficient directly affects the total cost incurred by the manufacturer, thereby indirectly influencing the pricing differences among the supply chain members under various decision-making frameworks.

**Proposition 7. **(i) $\frac{\partial \tau^{IC}}{\partial \delta }>0$;
(ii) $\tau^{IC}>\tau^{I}$.

See Appendix for proof.

**Fig 7:**
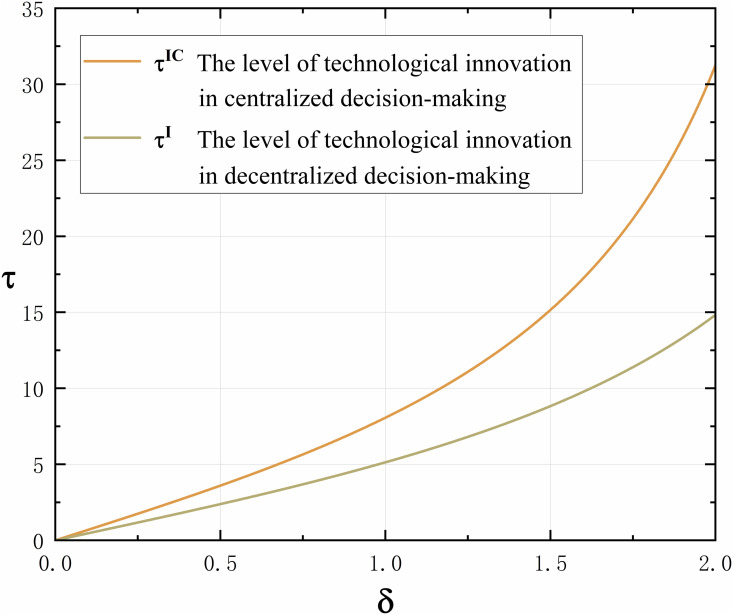
The trend of consumers’ technological innovation preference δ on technological innovation levels τ under centralized and decentralized decision-making.

Proposition 7 indicates that in centralized decision-making, the level of technological innovation increases with the increase of consumers’ technological innovation preference. According to Proposition 1, whether it is decentralized or centralized decision-making, consumers’ technological innovation preference has a positive impact on the level of technological innovation. After comparing centralized decision-making with decentralized decision-making, it can be seen that the level of technological innovation of manufacturer in centralized decision-making is greater than that in decentralized decision-making, and there is an interval that can be optimized and coordinated, as shown in Fig 7.

**Proposition 8.**(i) $\frac{\partial \prod^{IC}}{\partial \delta }>0$;
(ii) $\prod {\mathrm{\backslash nolimits}}^{IC}>\prod {\mathrm{\backslash nolimits}}^{I}$.

See Appendix for proof.

Proposition 8 indicates that consumers’ technological innovation preference in centralized decision-making can increase the profit of the whole supply chain. Based on Proposition 1, it can be concluded that whether it is decentralized or centralized decision-making, consumers’ technological innovation preference has a positive impact on the profit of the whole supply chain. After comparing the centralized and decentralized decision-making, it can be seen that the profit of whole supply chain in the centralized decision-making is greater than the decentralized decision-making, which can be further optimized and coordinated.

### 5.2 Revenue-sharing contract

**Fig 8:**
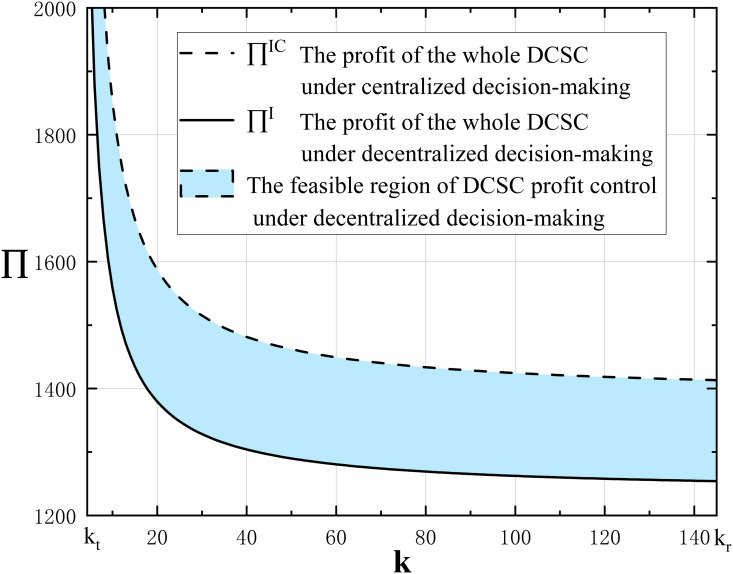
The trend of the technological innovation cost coefficient k on whole DCSC profit ∏ under centralized and decentralized decision-making.

This paper compares the DCSC model under the technological innovation mode in centralized and decentralized decision-making and concludes that the profit of the whole supply chain under centralized decision-making is greater than in decentralized decision-making, as shown in Fig 8. The shaded area shown in the figure indicates that there is a possibility of coordinated profits in the supply chain under decentralized decision-making, and the shaded area represents the feasible region of supply chain profits under decentralized decision-making. In order to improve the profit of the DCSC under decentralized decision-making, a revenue-sharing contract is used to enhance the profit of the whole DCSC under decentralized decision-making, achieving Pareto improvement in the profits of each member of the supply chain.

Due to space limitations, only the case of $k_{t}<k<k_{r}$ is considered in the revenue-sharing contract. Let $P_{r}^{Co}=P_{r}^{IC}$, $\tau^{Co}=\tau^{IC}$, $P_{t}^{Co}=P_{t}^{IC}$, and substitute them into equation (9) to calculate $W^{Co}$. Then, bring $P_{r}^{Co}$, $\tau^{Co}$, $P_{t}^{Co}$, $W^{Co}$ into the profit functions of the retailer, ECP, manufacturer, and the whole supply chain.

**Lemma 4.** In revenue-sharing contract, there exist equilibrium solutions and profits:

(i) The level of technological innovation

$$\tau^{Co}=\tau^{IC}$$

(ii) The profit of supply chain members

$$\prod {\mathrm{\backslash nolimits}}_{r}^{Co}=\frac{a^{2}k^{2}{\left(1-\gamma \right)}^{2}}{4{\left(k-\gamma k-\delta^{2}\right)}^{2}},$$

$$\prod {\mathrm{\backslash nolimits}}_{e}^{Co}=\frac{\lambda a^{2}k^{2}\left(1-\gamma \right)}{4{\left(k-\gamma k-\delta^{2}\right)}^{2}},$$

$$\prod {\mathrm{\backslash nolimits}}_{m}^{Co}=\frac{a^{2}\left(\left(1-\gamma \right)\left(\gamma +k-\lambda k\right)-2\delta^{2}\right)k}{4{\left(k-\gamma k-\delta^{2}\right)}^{2}},$$

(iii) The profit of the whole supply chain

$$\prod {\mathrm{\backslash nolimits}}^{Co}=\prod {\mathrm{\backslash nolimits}}^{C}=\frac{a^{2}k}{2\left(k-\gamma k-\delta^{2}\right)}$$

**Proposition 9.**(i) $\tau^{Co}>\tau^{I}$,
(ii) $\prod {\mathrm{\backslash nolimits}}_{r}^{Co}>\prod {\mathrm{\backslash nolimits}}_{r}^{I}$,
(ii) $\prod {\mathrm{\backslash nolimits}}_{m}^{Co}<\prod {\mathrm{\backslash nolimits}}_{m}^{I}$.

See Appendix for proof.

Proposition 9 indicates that after the occurrence of emergencies, the DCSC under the technological innovation mode fulfills the revenue-sharing contract, and both the level of technological innovation and retailer profits increase, but the manufacturer’s profits decrease compared to before coordination. This indicates that the revenue-sharing contract has not achieved Pareto improvement, and further design and improvement of the coordination contract are needed.

### 5.3 Improved revenue-sharing contract

Based on the in-depth analysis of the revenue-sharing contract in Section 5.2, we found that under the technological innovation mode, DCSC fails to achieve Pareto improvements during emergencies within the framework of a revenue-sharing contract. Therefore, drawing on the foundational models established by Zhou and Ye [70] as well as Xu et al. [71], we have refined the revenue-sharing contract. Because of the fact that the profits of the ECP come from commission provided by the manufacturer, it is easier for a manufacturer to form contractual relationships with an ECP. Therefore, we regard the manufacturer’s profit and the ECP’s profit as a whole, which is set to $\prod {\mathrm{\backslash nolimits}}_{em}$. The profit of the manufacturer and the ECP can be coordinated by adjusting the commission rate. To further design and improve the revenue-sharing contract, the manufacturer is compensated for the retailer’s increased profits in the revenue-sharing contract [72]. Let the compensation coefficient be β (0<β<1), then the increased profit of the retailer after coordination is $\Delta \prod {\mathrm{\backslash nolimits}}_{r}=\prod {\mathrm{\backslash nolimits}}_{r}^{Co}-\prod {\mathrm{\backslash nolimits}}_{r}^{I}$, the retailer’s profit is $\prod_{r}^{’}=\prod_{r}^{I}+(1-\beta )\Delta \prod_{r}$, and the total profit of the manufacturer and ECP after compensation is $\prod {\mathrm{\backslash nolimits}}_{em}^{’}=\prod {\mathrm{\backslash nolimits}}_{em}^{Co}+\beta \Delta \prod {\mathrm{\backslash nolimits}}_{r}$.

**Proposition 10.** If β’<β<1, the supply chain can achieve Pareto improvement.

See Appendix for proof.

**Fig 9:**
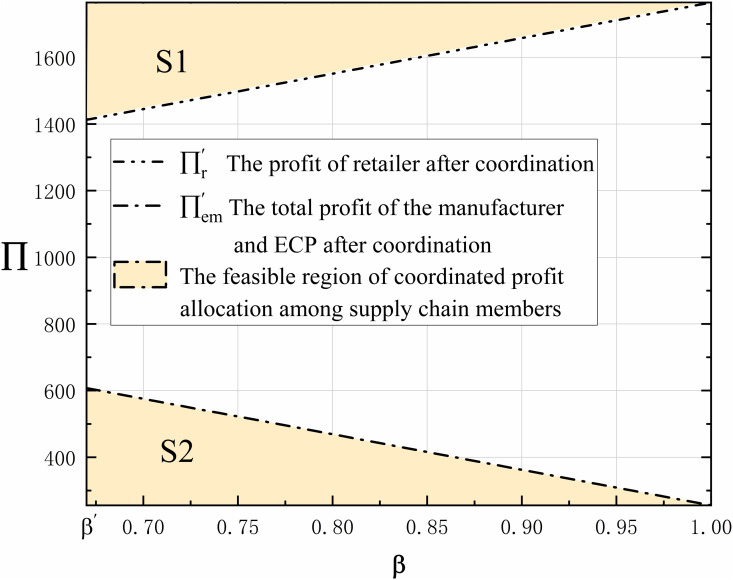
The trend of compensation coefficient β on the profits of coordinated DCSC members ∏.

**Fig 10:**
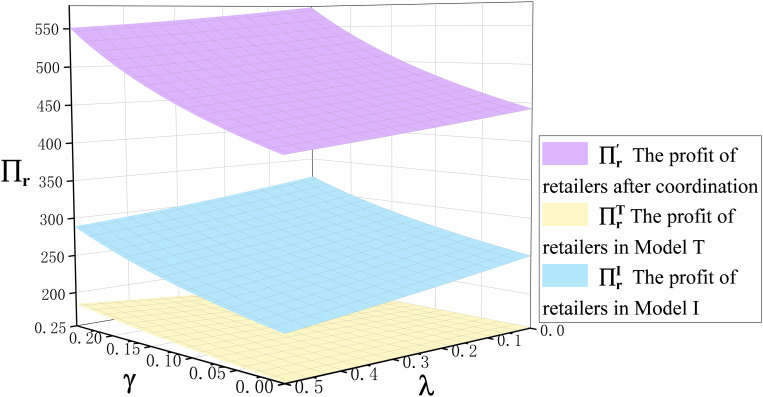
The trend of cross-price sensitivity coefficient γ and commission rate λ on retailer profits under different scenarios.

**Fig 11:**
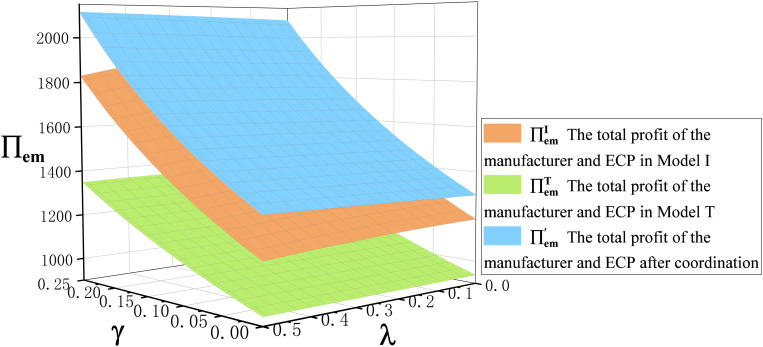
The trend of cross-price sensitivity coefficient γ and commission rate λ on the total profit of manufacturer and ECP $\prod {\mathrm{\backslash nolimits}}_{em}$ under different scenarios.

Proposition 10 shows that there exists a certain coordination interval (i.e., β∈(β’,1)) in the improved revenue-sharing contract where the supply chain can achieve Pareto improvement. As the coordination compensation coefficient increases, retailers compensate profits to manufacturers and ECPs, so the profits after coordination between manufacturers and ECPs gradually increase, as shown in Fig 9. Since the total profit increased by the supply chain remains the same after coordination, the two shaded areas in the figure are equal, i.e., S1 = S2. In addition, Fig 10. and Fig 11. fully verify that the improved revenue sharing constraint can achieve Pareto’s improvement in the supply chain.

In practice, take the sales of Huawei Mate 60 series smartphones as an example [73]. For sales channels, in the initial stage of sales, consumers need to pay a deposit in physical stores, but Huawei did not immediately open ECPs such as JD.com for sales. Huawei’s offline stores subsequently attracted a large number of consumers and earned substantial profits. Meanwhile, Luckin Coffee leverages high-frequency consumption at its offline stores to support the marginal cost of its “9.9 Yuan Coffee” online promotion [74]. This is consistent with the conclusion of this paper.

## 6 Conclusions

### 6.1 Findings

This article studies the pricing and innovation strategies of DCSC under traditional production mode and technological innovation mode in the event of emergencies. Through improved revenue-sharing contracts, supply chain members can increase profits in the event of emergencies, achieving Pareto improvement. In the event of an emergency, consumers tend to prefer technologically innovative products when shopping. Faced with changes in the external environment, it is an opportunity for manufacturers to choose technological innovation. However, technological innovation requires investment costs, and conservative manufacturers still choose to follow traditional production mode in the event of emergencies. Some manufacturers take advantage of unexpected events to cater to consumers’ technological innovation preferences and choose technological innovation mode. Therefore, we have established a DCSC mode consisting of manufacturer, retailer, and ECP under both traditional production mode and technological innovation mode. Parameters such as commission rate, cross-price elasticity coefficient, and consumers’ technological innovation preference are compared and analyzed for pricing and technological innovation strategies of supply chain members in traditional production mode and technological innovation mode. In addition, we found that the traditional revenue-sharing contract could not effectively coordinate the DCSC model under the technological innovation mode. Thus, we designed and improved the revenue-sharing contract. The results of the study show that:

First, during emergencies, when the manufacturer in a DCSC invests in technological innovation costs, both the demand and profits for the offline and e-commerce channels under the technological innovation mode exceed those observed under the traditional production mode.

Second, enhancing consumers’ technological innovation preference can increase the profit as well as the technological innovation level of the DCSC under emergencies. However, when the commission ratio of the ECP is large, it will hinder the improvement of the technological innovation level. In addition, the retail price and demand of the offline and ECP channels in the dual channel are affected by the commission rate. When the commission rate of the ECP is larger, the manufacturer will increase the retail price of the ECP, and then the demand for the ECP will decrease.

Finally, in the technological innovation mode after the occurrence of emergencies, traditional revenue-sharing contracts can improve the retailer’s profits and technological innovation level, but they cause losses to the manufacturer’s profits and cannot achieve Pareto improvement in the DCSC model. We have found through design and improvement that the improved revenue-sharing contract can ensure that the level of technological innovation is not affected and that the dual channel supply chain model achieves Pareto improvement under emergencies.

### 6.2 Managerial insights

The above research conclusions provide some management insights for supply chain members on pricing and coordination strategies for DCSC considering technological innovation in the event of emergencies.

The smooth operation of the supply chain may encounter a crisis when the external environment changes due to emergencies, but emergencies are not only a “challenge” to the supply chain, but also an “opportunity” for the supply chain. Manufacturers can adapt to the technological innovation preferences of consumers caused by unexpected events and actively invest in technological innovation costs to cater to consumers.

Under emergencies, government agencies should alleviate the cost burden of technological innovation on firms by streamlining patent application processes and expediting approvals for innovative enterprises, thereby reducing the external costs associated with technological innovation. Moreover, as a critical component of supply chain operations, ECPs should refrain from opportunistically increasing commission rates during such events. Instead, they should support manufacturers in their transition toward technological innovation, thereby fostering a win-win scenario across the entire supply chain.Under emergencies, the DCSC should avoid vicious competition among channels, and cooperation among supply chain members is more conducive to enhancing the profit and technological innovation level of each member when the traditional revenue-sharing contract cannot effectively enhance the supply chain members’ profit, it can be based on this paper to coordinate the members of the DCSC with the designed and improved revenue-sharing contract.

### 6.3 Further research

This paper focuses on analyzing the pricing and coordination strategies of a DCSC that incorporates technological innovation during emergencies, while not addressing the manufacturer’s financial issues. Future research could investigate whether manufacturers possess sufficient funds for technological innovation during emergencies and explore financing and innovation investment strategies under capital constraints for DCSC, thereby providing more optimal strategic choices for all supply chain members.

## Supporting information

S1 Appendix Proof of proposition. (DOCX)

S2 File Excel spreadsheet of data from articles. (ZIP)
