# Supplementary material for: Should manufacturers choose technological innovation in dual-channel supply chains during emergencies?
Source: PLoS One. 2025 Jul 3;20(7):e0327014. doi: 10.1371/journal.pone.0327014 (PMC12225839; doi:10.1371/journal.pone.0327014)
Supplement: S1 Appendix — (DOCX) [file pone.0327014.s001.docx]

# Appendix

**1. Proof of Proposition 1.**

**Subject to** $0<\gamma<\frac{1}{4}$, $k>\frac{\delta^{2}}{1-\gamma}$ **and** $0<\lambda<\frac{1}{2}$**, we can derive that**

(i)$\frac{\partial W^{I}}{\partial\delta}=\frac{4a\delta k\lambda_{1}\psi_{1}(4\gamma_{2}-2\gamma\lambda-\gamma^{2}\lambda)}{(\psi_{2}k-2\psi_{1}\delta^{2})^{2}}>0$,

$\frac{\partial P_{r}^{I}}{\partial\delta}=\frac{4a\delta k\lambda_{1}\psi_{1}((4\gamma_{2}+2\gamma_{1}\gamma_{2}-\gamma\lambda)}{(\psi_{2}k-2\psi_{1}\delta^{2})^{2}}>0$,

$\frac{\partial P_{e}^{I}}{\partial\delta}=\frac{4a\delta k\psi_{1}(4\lambda_{1}+4\gamma-3\gamma\lambda)}{(\psi_{2}k-2\psi_{1}\delta^{2})^{2}}>0$.

(ii)$\frac{\partial\tau^{I}}{\partial\delta}=\frac{2\psi_{1}a(\psi_{2}k+2\psi_{1}\delta^{2})}{(\psi_{2}k-2\psi_{1}\delta^{2})^{2}}>0$,

(iii)$\frac{\partial D_{r}^{I}}{\partial\delta}=\frac{4a\delta k\gamma_{2}\lambda_{1}\psi_{1}(2\gamma_{1}+\gamma\lambda)}{(\psi_{2}k-2\psi_{1}\delta^{2})^{2}}>0$,

$\frac{\partial D_{e}^{I}}{\partial\delta}=\frac{4a\delta k\gamma_{2}\psi_{1}(4\lambda_{1}-2\lambda_{1}\gamma^{2}-2\gamma+\gamma\lambda)}{(\psi_{2}k-2\psi_{1}\delta^{2})^{2}}>0$;

(iv)$\frac{\partial\prod_{r}^{I}}{\partial\delta}=\frac{8Aa^{2}k^{2}\lambda_{1}^{2}\gamma_{2}^{2}(\gamma\lambda+2\gamma_{1})^{2}\delta}{(\psi_{2}k-2\psi_{1}\delta^{2})^{3}}>0$,

$\frac{\partial\prod_{e}^{I}}{\partial\delta}=\frac{8\psi_{1}a^{2}k^{2}\lambda\gamma_{2}(4\lambda_{1}+\gamma\lambda-2\gamma-2\gamma^{2}\lambda_{1})(4\gamma+4\lambda_{1}-3\gamma\lambda)\delta}{(\psi_{2}k-2\psi_{1}\delta^{2})^{3}}>0$,

Taking $\frac{\partial\prod_{e}^{I}}{\partial\delta}=\frac{8\psi_{1}a^{2}k^{2}\lambda\gamma_{2}(4\lambda_{1}+\gamma\lambda-2\gamma-2\gamma^{2}\lambda_{1})(4\gamma+4\lambda_{1}-3\gamma\lambda)\delta}{(\psi_{2}k-2\psi_{1}\delta^{2})^{3}}>0$ as an example, we first perform factorization on the expression to the greatest extent possible and subsequently analyze the sign of each component. Subject to $0<\gamma<\frac{1}{4}$, $k>\frac{\delta^{2}}{1-\gamma}$ and $0<\lambda<\frac{1}{2}$, we can derive that $\psi_{2}>0$. Consequently, $\psi_{2}k-2\psi_{1}\delta^{2}$ is an increasing function with respect to $k$. By substituting$k=\frac{\delta^{2}}{1-\gamma}$into$\psi_{2}k-2\psi_{1}\delta^{2}$, we obtain $\psi_{2}k-2\psi_{1}\delta^{2}>0$, which further implies that the denominator$(\psi_{2}k-2\psi_{1}\delta^{2})^{3}>0$. In the molecule, $8\psi_{1}a^{2}k^{2}\lambda\gamma_{2}\delta>0$, $4\lambda_{1}+\gamma\lambda-2\gamma-2\gamma^{2}\lambda_{1}>0$，$4\gamma+4\lambda_{1}-3\gamma\lambda>0$， So, $\frac{\partial\prod_{e}^{I}}{\partial\delta}=\frac{8\psi_{1}a^{2}k^{2}\lambda\gamma_{2}(4\lambda_{1}+\gamma\lambda-2\gamma-2\gamma^{2}\lambda_{1})(4\gamma+4\lambda_{1}-3\gamma\lambda)\delta}{(\psi_{2}k-2\psi_{1}\delta^{2})^{3}}>0$.

$\frac{\partial\prod_{e}^{I}}{\partial\delta}=\frac{8\psi_{1}a^{2}k^{2}\lambda\gamma_{2}(4\lambda_{1}+\gamma\lambda-2\gamma-2\gamma^{2}\lambda_{1})(4\gamma+4\lambda_{1}-3\gamma\lambda)\delta}{(\psi_{2}k-2\psi_{1}\delta^{2})^{3}}>0$,

$\frac{\partial\prod_{m}^{I}}{\partial\delta}=\frac{4(\psi_{1})^{2}a^{2}k\delta}{(\psi_{2}k-2\psi_{1}\delta^{2})^{2}}>0$,

$\frac{\partial\prod^{I}}{\partial\delta}=\frac{\begin{matrix} 4\psi_{1}a^{2}k\delta((\gamma^{3}\lambda^{4}+(16\gamma_{2}-14\gamma^{3}-5^{2})\lambda^{3}+\gamma(29\gamma^{2}-13\gamma-32)\lambda^{2}-16\gamma_{1}\gamma_{2}(3-\gamma)\lambda\\ +32\gamma_{1}\gamma_{2})k\gamma_{2}-2(\psi_{1})^{2}\delta^{2} \end{matrix}}{(\psi_{2}k-2\psi_{1}\delta^{2})^{3}}>0$.

**2. Proof of Proposition 2.**

**Subject to** $0<\gamma<\frac{1}{4}$, $k>\frac{\delta^{2}}{1-\gamma}$ **and** $0<\lambda<\frac{1}{2}$**, we can derive that**

(i) $\frac{\partial\tau^{I}}{\partial\lambda}=-\frac{2ak(4\lambda_{1}+\gamma\lambda-2\gamma-2\gamma^{2}\lambda_{1})(4\gamma+4\lambda_{1}-3\gamma\lambda)\delta}{(\psi_{2}k-2\psi_{1}\delta^{2})^{2}}<0$,

(ii) $P_{r}^{I}-P_{e}^{I}=\frac{ak(\gamma\lambda^{2}+2(\gamma^{2}-\gamma-1)\lambda-2\gamma^{2}+2)}{\psi_{2}k-2\psi_{1}\delta^{2}}$, $P_{r}^{I}-P_{e}^{I}$is a quadratic function about $\lambda$. There exist two real roots $\lambda_{P1}^{I}=\frac{\gamma+\gamma_{1}\gamma_{2}-\sqrt{\gamma_{1}\gamma_{2}+\gamma^{4}}}{\gamma}$ and $\lambda_{P2}^{I}=\frac{\gamma+\gamma_{1}\gamma_{2}+\sqrt{\gamma_{1}\gamma_{2}+\gamma^{4}}}{\gamma}>1$. Therefore, if $0<\lambda<\lambda_{P1}^{I}$, $P_{r}^{I}>P_{e}^{I}$; if $\lambda_{P1}^{I}<\lambda<1$, $P_{e}^{I}>P_{r}^{I}$.

(iii) $D_{r}^{I}-D_{e}^{I}=-\frac{ak\gamma_{2}(\gamma\lambda^{2}+2\gamma_{1}\gamma_{2}\lambda_{1}-2\gamma\lambda)}{\psi_{2}k-2\psi_{1}\delta^{2}}$. The proof of (3) is similar to that of (2), thus, we omit it.

**3. Proof of Proposition 3.**

**Subject to** $0<\gamma<\frac{1}{4}$, $k>\frac{\delta^{2}}{1-\gamma}$ **and** $0<\lambda<\frac{1}{2}$**, we can derive that**

(i) $W^{I}-W^{T}=\frac{2a\psi_{1}\lambda_{1}(4\gamma_{2}-(2+\gamma)\gamma\lambda)\delta^{2}}{\psi_{2}(\psi_{2}k-2\psi_{1}\delta^{2})}>0$,

(ii) $P_{r}^{I}-P_{r}^{T}=\frac{2a\psi_{1}\lambda_{1}(4\gamma_{2}+2\gamma_{1}\gamma_{2}-\gamma\lambda)\delta^{2}}{\psi_{2}(\psi_{2}k-2\psi_{1}\delta^{2})}>0$,

(iii) $P_{e}^{I}-P_{e}^{T}=\frac{2a\psi_{1}\lambda_{1}(4\lambda_{1}+4\gamma-3\gamma\lambda)\delta^{2}}{\psi_{2}(\psi_{2}k-2\psi_{1}\delta^{2})}>0$;

**4. Proof of Proposition 4.**

**Subject to** $0<\gamma<\frac{1}{4}$, $k>\frac{\delta^{2}}{1-\gamma}$ **and** $0<\lambda<\frac{1}{2}$**, we can derive that**

(i) $D_{r}^{I}-D_{r}^{T}=\frac{2a\psi_{1}\gamma_{2}\lambda_{1}(2\gamma_{1}-\gamma\lambda)\delta^{2}}{\psi_{2}(\psi_{2}k-2\psi_{1}\delta^{2})}>0$,

(ii) $D_{e}^{I}-D_{e}^{T}=\frac{2a\psi_{1}\gamma_{2}(\lambda_{1}(4-2\gamma^{2})-2\gamma+\gamma\lambda)\delta^{2}}{\psi_{2}(\psi_{2}k-2\psi_{1}\delta^{2})}>0$;

**5. Proof of Proposition 5.**

**Subject to** $0<\gamma<\frac{1}{4}$, $k>\frac{\delta^{2}}{1-\gamma}$ **and** $0<\lambda<\frac{1}{2}$**, we can derive that**

(i) $\prod_{r}^{I}-\prod_{r}^{T}=\frac{4a^{2}\psi_{1}\gamma_{2}^{2}\lambda_{1}^{2}(2\gamma_{1}+\gamma\lambda)(\psi_{2}k-\psi_{1}\delta^{2})\delta^{2}}{(\psi_{2})^{2}(\psi_{2}k-2\psi_{1}\delta^{2})^{2}}>0$,

(ii) $\prod_{e}^{I}-\prod_{e}^{T}=\frac{4a^{2}\psi_{1}\gamma_{2}\lambda(4\lambda_{1}+\gamma\lambda-2\gamma-2\gamma^{2}\lambda_{1})(4\gamma+4\lambda_{1}-3\gamma\lambda)(\psi_{2}k-\psi_{1}\delta^{2})\delta^{2}}{(\psi_{2})^{2}(\psi_{2}k-2\psi_{1}\delta^{2})^{2}}>0$,

(iii) $\prod_{m}^{I}-\prod_{m}^{T}=\frac{2a^{2}(\psi_{1})^{2}\delta^{2}}{\psi_{2}(\psi_{2}k-2\psi_{1}\delta^{2})}>0$.

**6. Proof of Proposition 6.**

**Subject to** $0<\gamma<\frac{1}{4}$, $k>\frac{\delta^{2}}{1-\gamma}$ **and** $0<\lambda<\frac{1}{2}$**, we can derive that**

$P_{r}^{IC}-P_{r}^{I}=-\frac{ak((2\lambda_{1}(2\gamma_{1}-\gamma\lambda+2\gamma^{3})-(4-6\lambda+\lambda^{2})\gamma^{2})k-2\lambda_{1}(3\gamma_{1}\gamma_{2}+(\gamma_{2}+\gamma^{2}))\delta^{2})}{2(\gamma_{1}k-\delta^{2})(\psi_{2}k-2\psi_{1}\delta^{2})}$,

$P_{r}^{IC}-P_{r}^{I}$ is a quadratic function about $k$. There exist two real roots 0 and $k_{t}$,

$k_{t}=\frac{2\lambda_{1}(3\gamma_{1}\gamma_{2}\text{+}2\lambda\gamma_{2}+\lambda\gamma^{2})\delta^{2}}{4\lambda_{1}(1+\gamma^{3})-(\lambda^{2}-6\lambda+4)\gamma^{2}-2\lambda_{1}(1+\lambda_{2})\gamma}$. Similarly, $P_{e}^{IC}-P_{e}^{I}=-\frac{ak((\gamma^{2}\lambda^{2}+2\gamma\gamma_{1}\lambda)k+2((2+\gamma)\gamma_{2}\lambda^{2}-(2\gamma^{2}+4\gamma+1)\lambda-\gamma_{1}\gamma_{2})\delta^{2})}{2(\gamma_{1}k-\delta^{2})(\psi_{2}k-2\psi_{1}\delta^{2})}$, $P_{e}^{IC}-P_{e}^{I}$ is a quadratic function about $k$. There exist two real roots 0 and $k_{r}$, $k_{r}=\frac{2(\lambda_{2}-\gamma^{2}\lambda_{1}^{2}-2\lambda^{2}+2\gamma\lambda-3\gamma\lambda^{2})\delta^{2}}{\gamma\lambda(\gamma\lambda+2\gamma_{1})}$. In addition, $k_{r}-k_{t}=\frac{2((3\lambda^{2}-2\lambda_{2})\gamma^{2}-2\gamma\gamma_{1}\gamma_{2}\lambda_{1}^{2}-2(\lambda^{2}-\lambda_{2}))(\gamma\lambda^{2}+2(\gamma^{2}-\gamma_{2})\lambda+2\gamma_{1}\gamma_{2})\delta^{2}}{\gamma\lambda(\gamma\lambda+2\gamma_{1})(4\lambda_{1}(1+\gamma^{3})-(\lambda^{2}-6\lambda+4)\gamma^{2}-2\gamma\lambda_{1}(2+\lambda))}>0$,$k_{t}-k_{0}=\frac{((3\lambda^{2}-2\lambda_{2})\gamma^{2}-2\gamma\gamma_{1}\gamma_{2}\lambda_{1}^{2}-2(\lambda^{2}-\lambda_{2}))\delta^{2}}{\gamma_{1}(4\lambda_{1}(1+\gamma^{3})-(\lambda^{2}-6\lambda+4)\gamma^{2}-2\gamma\lambda_{1}(2+\lambda))}>0$. Therefore, $k_{0}<k_{t}<k_{r}$.

**7. Proof of Proposition 7.**

**Subject to** $0<\gamma<\frac{1}{4}$, $k>\frac{\delta^{2}}{1-\gamma}$ **and** $0<\lambda<\frac{1}{2}$**, we can derive that**

(i) $\frac{\partial\tau^{IC}}{\partial\delta}=\frac{a(\gamma_{1}k+\delta^{2})}{(\gamma_{1}k-\delta^{2})^{2}}>0$**;**

(ii) $\tau^{IC}-\tau^{I}=\frac{ak(2\gamma_{1}\gamma_{2}(\lambda_{2}-\gamma\lambda_{1}^{2})+3\gamma^{2}\lambda^{2}-4\lambda^{2})\delta}{(\gamma_{1}k-\delta^{2})(\psi_{2}k-2A\delta^{2})}>0$.

**8. Proof of Proposition 8.**

**Subject to** $0<\gamma<\frac{1}{4}$, $k>\frac{\delta^{2}}{1-\gamma}$ **and** $0<\lambda<\frac{1}{2}$**, we can derive that**

(i) $\frac{\partial\prod^{IC}}{\partial\delta}=\frac{a^{2}k\delta}{(\gamma_{1}k-\delta^{2})^{2}}>0$;

(ii) $\prod^{IC}-\prod^{I}=\frac{\begin{matrix} a^{2}k^{2}((8(\gamma_{1}-\gamma\lambda)\lambda_{1}^{2}+2\gamma^{2}(\lambda^{4}-8\lambda_{1}^{2})(2-\gamma^{2})+4(4\lambda_{1}-3\lambda^{2}\lambda_{1})\gamma^{3}-4\lambda_{1}(2-\lambda^{2})\gamma^{5})k-2\delta^{2}\gamma_{2}(2\lambda_{1} \\ -10\lambda\lambda_{1}+8\lambda^{2}\lambda_{1}(\lambda-\gamma\lambda_{1})+(3\lambda^{4}+2\lambda^{3}+20\lambda\lambda_{1}-4)\gamma^{2}+2(4-3\lambda)\lambda^{2}\lambda_{1}\gamma^{3}+2\lambda_{1}^{4}\gamma^{4})) \end{matrix}}{2(\gamma_{1}k-\delta^{2})(\psi_{2}k-2\psi_{1}\delta^{2})^{2}}>0$

**9. Proof of Proposition 9.**

**Subject to** $0<\gamma<\frac{1}{4}$, $k>\frac{\delta^{2}}{1-\gamma}$ **and** $0<\lambda<\frac{1}{2}$**, we can derive that**

(i) $\tau^{Co}-\tau^{I}=\frac{ak(2\gamma_{1}\gamma_{2}(\lambda_{2}-\gamma\lambda_{1}^{2})+3\gamma^{2}\lambda^{2}-4\lambda^{2})\delta}{(\gamma_{1}k-\delta^{2})(\psi_{2}k-2A\delta^{2})}>0$

(ii) $\prod_{t}^{Co}-\prod_{t}^{I}=\frac{\begin{matrix} a^{2}k^{2}((\gamma_{1}(4-2\lambda-\lambda^{2})\gamma^{2}-\lambda_{1}(4-2\lambda\gamma))k+2(1-2\lambda-\lambda_{1})\gamma^{2})\gamma_{2}\lambda_{1}\eta^{2})(((12 \\ -14\lambda+3\lambda^{2})\gamma^{2}-2\lambda_{1}(6+\lambda\gamma))\gamma_{1}k+2(5-2\lambda-2(2-\lambda)\gamma-\lambda_{1}\gamma^{2})\gamma_{2}\lambda_{1}\delta^{2}) \end{matrix}}{4(\gamma_{1}k-\delta^{2})^{2}(\psi_{2}k-2\psi_{1}\delta^{2})^{2}}$,

is a quadratic concave function about $k$. There exist two real roots $k_{t1}^{Co}$ and $k_{t2}^{Co}$, $k_{t1}^{Co}=\frac{2(5-2\lambda-2(2-\lambda)\gamma-\lambda_{1}\gamma^{2})\gamma_{2}\lambda_{1}\delta^{2}}{-\gamma_{1}((3\lambda^{2}-14\lambda+12)\gamma^{2}-2\lambda_{1}(6+\gamma\lambda))}$, $k_{t2}^{Co}=\frac{2(1-2\lambda-\lambda_{1}\gamma^{2})\gamma_{2}\lambda_{1}\delta^{2}}{\gamma_{1}((\lambda^{2}+2\lambda-4)\gamma^{2}-2\lambda_{1}(\gamma\lambda-2))}$. $k_{t}-k_{t1}^{Co}=\frac{4\lambda_{1}(\gamma_{1}\gamma_{2}(4-\gamma)+\gamma^{2}\lambda)(2\lambda_{1}^{2}\gamma^{3}+(3\lambda^{2}-2\lambda_{2})\gamma^{2}-2\lambda_{1}^{2}\gamma-4\lambda^{2}+2\lambda_{2})\delta^{2}}{\gamma_{1}(-4\gamma_{1}\gamma^{3}+(\lambda^{2}-6\lambda+4)\gamma^{2}-2(\lambda\lambda_{2}-2)\gamma-4\lambda_{1})((3\gamma^{2}+2\gamma)\lambda^{2}-2(1-6\gamma_{1})\gamma_{2}\lambda-12\gamma_{1}\gamma_{2})}>0$ $k_{t}-k_{t2}^{Co}=\frac{4\lambda_{1}(\gamma^{3}-\gamma\lambda_{2}-2\gamma_{1}\gamma_{2})(2\lambda_{1}^{2}\gamma^{3}+(3\lambda^{2}-2\lambda_{2})\gamma^{2}-2\lambda_{1}^{2}\gamma-4\lambda^{2}+2\lambda_{2})\delta^{2}}{\gamma_{1}(4\gamma_{1}\gamma^{3}-(\lambda^{2}-6\lambda+4)\gamma^{2}+2(\lambda\lambda_{2}-2)\gamma+4\lambda_{1})((\gamma^{2}+2\gamma)\lambda^{2}-2(2-\gamma)\gamma_{2}\lambda+4\gamma_{1}\gamma_{2})}>0$.Therefore, within $k_{t}<k<k_{r}$, there is always .

(iii) $\prod_{m}^{Co}-\prod_{m}^{I}=\frac{\begin{matrix} a^{2}k^{2}(((3\lambda^{2}-4)\gamma^{2}+\lambda_{1}(4-\lambda^{2})-4\lambda_{1}(1-\lambda_{2}\gamma))\gamma_{1}k+2\eta^{2}(\gamma_{1}\gamma_{2}(2+\gamma)\lambda^{3}+(\gamma^{3} \\ -\gamma^{2}\gamma_{1}\gamma_{2}+\gamma_{1})\lambda^{2}-2\gamma_{1}\gamma_{2}(2-\gamma^{2})\lambda+\gamma_{1}^{2}\gamma_{2}^{2})) \end{matrix}}{4(k-\gamma k-\eta^{2})^{2}(\psi_{2}k-2\psi_{1}\delta^{2})}$. The proof of (3) is similar to that of (2), thus, we omit it.

**10. Proof of Proposition 10.**

**Subject to** $0<\gamma<\frac{1}{4}$, $k>\frac{\delta^{2}}{1-\gamma}$ **and** $0<\lambda<\frac{1}{2}$**, we can derive that**

If the DCSC achieves Pareto improvement, it needs to meet ,. That is $\frac{\prod_{em}^{I}-\prod_{em}^{Co}}{\prod_{r}^{Co}-\prod_{r}^{I}}<\beta<1$. Let $\frac{\prod_{em}^{I}-\prod_{em}^{Co}}{\prod_{r}^{Co}-\prod_{r}^{I}}=\beta’$, The calculation gives $\beta’=\frac{\gamma_{1}\gamma_{2}(\gamma\lambda-2\gamma_{1})Q_{1}k^{2}-2\eta^{2}Q_{2}k+4\gamma_{2}\eta^{4}Q_{3}}{\begin{matrix} (\gamma_{1}((\lambda^{2}+2\lambda-4)\gamma^{2}-2\lambda\lambda_{1}\gamma+4\lambda_{1})k+2\lambda_{1}\gamma_{2}(\lambda_{1}\gamma^{2}+2\lambda-1)\eta^{2})(\gamma_{1}(-(3\gamma^{2}+2\gamma)\lambda^{2}-2(7\gamma\gamma_{1} \\ +6\gamma_{2})\lambda+12\gamma_{1}\gamma_{2})k+2\lambda_{1}\gamma_{2}(\lambda_{1}\gamma^{2}+2(2-\lambda)\gamma+2\lambda-5)\eta^{2}) \end{matrix}}$

where:

$$Q_{1}=(3\lambda^{3}+26\lambda^{2}-24\lambda+16)\gamma^{3}+2(2\lambda^{3}-11\lambda^{2}+16\lambda-8)\gamma^{2}-8(\lambda^{3}-3\lambda+2)\gamma+16\lambda_{1}^{2}$$

$$\begin{matrix} Q_{2}=2(\lambda^{2}+4\lambda-6)\lambda_{1}^{2}\gamma^{6}+2(7\lambda^{4}-13\lambda^{3}-2\lambda^{2}+12\lambda-4)\gamma^{5}+(13\lambda^{4}-66\lambda^{3}+132\lambda^{2}-120\lambda+44)\gamma^{4}-2(15\lambda^{4}-25\lambda^{3} \\ -6\lambda^{2}+24\lambda+8)\gamma^{3}-2(16\lambda^{4}-55\lambda^{3}+87\lambda^{2}-72\lambda+26)\gamma^{2}+8(2\lambda^{4}-3\lambda^{3}-\lambda^{2}+3\lambda-1)\gamma+\lambda_{1}^{2}(5-4\lambda\lambda_{1}) \end{matrix}$$

$$\begin{matrix} Q_{3}=\lambda_{1}^{4}\gamma^{5}+(3-\lambda)\lambda_{1}^{3}\gamma^{4}-2(4\lambda^{4}-11\lambda^{3}+10\lambda^{2}-4\lambda+1)\gamma^{3}-(11\lambda^{4}-34\lambda^{3}+38\lambda^{2}-20\lambda+6)\gamma^{2}+(8\lambda^{4}-18\lambda^{3}+13\lambda^{2} \\ -4\lambda+1)\gamma+12\lambda^{4}-28\lambda^{3}+23\lambda^{2}-10\lambda+3 \end{matrix}$$

$\gamma_{1}=1-\gamma$, $\gamma_{2}=1+\gamma$;

$\lambda_{1}=1-\lambda$, $\lambda_{2}=1+\lambda$;

$\psi_{1}=\gamma_{2}\lambda_{1}(\gamma_{2}+2\lambda_{1}-\gamma\lambda)$, $\psi_{2}=8\gamma_{1}\gamma_{2}\lambda_{1}-\gamma^{2}\lambda^{2}$.
